# Cases Showing the Advantage of Heroic Doses of Calomel

**Published:** 1858-05

**Authors:** Wm. Hauser

**Affiliations:** Jefferson county, Georgia


### ARTICLL III.

*Cases showing the advantage of heroic doses of Calomel.* By WM. HAUSER, M. D., of Jefferson County, Ga.

An experience of fourteen years, has convinced me that no one thing, nor all other things combined, are equal to Calomel *in Herculean doses*, for the treatment of acute gastritis and bilious colic. Without any waste of words, I give a few cases: First premising that my range of practice lies in that beautiful long-leaf pine, and sand belt of Georgia, near 33d parallel of North latitude, which is checkered alternately with high and dry pine barrenns, celebrated for their healthfulness, and rich alluvial "oak and hickory" lands, where hepatic derangements are found in plenty.

CASE 1st. Wm. Caruthers, of Burke county, aged about 30, tolerably plethoric and of steady habits, when I knew him, was attacked with symptoms of acute gastritis, most violent, October, 1843. Venesection was attempted but failed. The inflammation appeared to progress more rapidly than in any case I have ever noticed before or since. Having no time to trifle with doubtful medicines, I instantly poured down him 40 grains of Calomel and the same of Jalap. He retained it, and in a few hours I repeated the dose. In twelve hours he was happily convalescent, and soon got well.

CASE II. James Wooten, aged about 35, of robust frame and vigorous constitution. Called to him on Monday, May 6th, 1844. He was excessively bilious; looked as though his liver might have failed in its functions a month previous. Suffering with symptoms of acute gastritis, I premised venesection, and gave 85 grains of calomel. It operated in a few hours, and he was quite relieved. The second day he rode out, and seemed entirely cured.

CASE III. Mrs. Lucinda Bryant, of Washington County. I was called to her in consultation with my esteemed friend, Dr. Wm. B. Folks, on the 15th of October, 1855. Her stomach would retain nothing. She was suffering most terribly. My friend had tried everything he could think of, and even calomel itself, but in doses too small to remain in the stomach. I administered, without the least hesitation, ʒi. of this article. She retained it, was relieved in ten hours, and her recovery was rapid. She had been suffering two or three days with gastritis, and death seemed inevitable under any circumstances. Calomel is one of the finest anti-septics in the world, as I have found by a great multitude of experiments, although

Dr. Dunglison does not even mention it as one of the chief articles of that class. I believe its virtues are not fully known. Every body has learned something of its cathartic and febrifuge properties, and its very commonness in the hands of physicians has induced a carelessness about studying its properties and watching its effects. New remedies are the rage, while some of the older and more valuable are treated with unjust neglect, to the detriment of Doctors and their patients.

CASE IV. Miss Julia Parker, of Jefferson County,'185–, case of remittent fever, with frequent vomitings and inability to retain any medicine except calomel. A large dose of this produced immediate quiet, and prepared the way for a speedy recovery.

CASE V. Myself. Called on the 16th October, 1854, to perform a journey of 25 miles, to consult with my friend, Dr. Wm. G. Allen, over a dying lady. My accustomed gastralgia, came on by the time I had made about three-fifths of the distance, and the jolting of the buggy and vomiting for the remainder of the way, developed symptoms of gastritis, and such excessive irritability of stomach, that the gentlest shake of the bed would bring on convulsions horrible to behold. Bleeding, injections, sinapisms, all failed to bring sufficient quiet to the stomach to enable it to retain any thing that was swallowed. At last I asked my friends to give me a tea-spoonful of Calomel. This remained only a few minutes. I then requtsted a table spoonful. That remained and checked the vomiting. In thirty-six hours it operated and I was relieved.

I might encumber the *Journal* with a large number of cases; but these are deemed sufficient to show the importance of giving calomel in heroic doses to patients suffering from gastritis. And the same holds good in bilious colic wherein bile is vomited in a tide, and the bowels so constricted that no operation can be induced by injections, hot baths, or any medicines that may be given, save calomel. But it must be given in *large doses* to bring speedy and full relief; and there is not the least danger in so administering it. I have in a few cases, been called long distances to consult with young physicians, who, I found, were pursuing the right plan of treatment, saving their timidity in administering calomel. In such cases I have only had to nerve them up to bolder doings, and success has always followed.

